# Novel Co-Cultivation Bioprocess with Immobilized *Paenibacillus polymyxa* and *Scenedesmus obliquus* for Lipid and Butanediol Production

**DOI:** 10.3390/microorganisms13030606

**Published:** 2025-03-05

**Authors:** Jnanada Shrikant Joshi, Laura Fladung, Olaf Kruse, Anant Patel

**Affiliations:** 1 Bielefeld Institute of Applied Materials Research, Hochschule Bielefeld—University of Applied Sciences and Arts, 33619 Bielefeld, Germany; jnanada_shrikant.joshi@hsbi.de (J.S.J.); laura.fladung@hsbi.de (L.F.); 2 Faculty of Biology, Bielefeld University, 33615 Bielefeld, Germany; olaf.kruse@uni-bielefeld.de

**Keywords:** bacterium, microalga, immobilization, co-cultivation, lipid production, biomass

## Abstract

Microalgal biotechnology is gaining attention due to its potential to produce pigments, lipids, biofuels, and value-added products. However, challenges persist in terms of the economic viability of microalgal lipid production in photobioreactors due to slow growth rates, expensive media, complex downstream processing, limited product yields, and contamination risks. Recent studies suggest that co-cultivating microalgae with bacteria can enhance the profitability of microalgal bioprocesses. Immobilizing bacteria offers advantages such as protection against shear forces, the prevention of overgrowth, and continuous product secretion. Previous work has shown that biopolymeric immobilization of *Paenibacillus polymyxa* enhances 2,3-butanediol production. In this study, a novel co-fermentation process was developed by exploiting the chemical crosstalk between a freshwater microalga *Scenedesmus obliquus*, also known as *Tetradesmus obliquus*, and an immobilized plant-growth-promoting bacterium, *Paenibacillus polymyxa*. This co-cultivation resulted in increased metabolite production, with a 1.5-fold increase in the bacterial 2,3-butanediol concentration and a 3-fold increase in the microalgal growth rates compared to these values in free-cell co-cultivation. Moreover, the co-culture with the immobilized bacterium exhibited a 5-fold increase in the photosynthetic pigments and a 3-fold increase in the microalgal lipid concentration compared to these values in free-cell co-cultivation. A fixed bed photobioreactor was further constructed, and the co-cultivation bioprocess was implemented to improve the bacterial 2,3-butanediol and microalgal lipid production. In conclusion, this study provides conclusive evidence for the potential of co-cultivation and biopolymeric immobilization techniques to enhance 2,3-butanediol and lipid production.

## 1. Introduction

Microalgal biotechnology has emerged as a promising avenue for producing biofuels and lipids, exploring the potential of microalgae as sustainable sources of renewable energy and valuable lipids [1]. One significant advantage of microalgal biotechnology lies in the high lipid content of many microalgal species; these lipids can be converted into biofuels [2]. Microalgae can thrive in diverse environments, including saline water and wastewater, minimizing competition with food crops for arable land [3]. Additionally, microalgae can capture carbon dioxide from industrial flue gases or wastewater, providing a sustainable solution for carbon sequestration while mitigating greenhouse gas emissions [4]. Moreover, microalgae offer a potential solution for green energy, as they require minimal land and utilize various nutrient sources compared to traditional oilseed crops [5,6]. In addition to their high biomass production, microalgae have a relatively high photosynthetic efficiency in fixing CO_2_ [7,8,9]. An example of a promising production strain is the green algae genus *Scenedesmus* [10,11]. *Scenedesmus* species produce secondary plant substances, such as carotenoids, that act as photoprotective agents, protecting chlorophyll molecules or cells from the destruction caused by photooxidation [11]. In addition to producing pigments, the genus *Scenedesmus* can synthesize large amounts of long-chain fatty acids [12]. Matsunaga et al. demonstrated *Scenedesmus* lipid accumulation of up to 73%, which could be utilized to produce biofuels [13]. It has been reported that *Tetradesmus obliquus*, previously known as *Scenedesmus obliquus*, significantly synthesizes and accumulates lipids such as triacylglycerol (TAG) and carbohydrates [14,15]. Thus, *T. obliquus*, referred to herein as *S. obliquus*, can be considered a model organism for lipid production.

However, microalgal biotechnology also presents significant challenges. Scaling up microalgal cultivation to commercial levels remains economically challenging due to the high production costs of cultivation, harvesting, and lipid extraction processes [1]. Additionally, there are technical hurdles to consistent lipid productivity under varying environmental conditions that still require further optimization. Different biomass feedstocks, fluctuating costs, and regulatory constraints also result in additional hurdles to the widespread adoption of microalgal biofuels [4]. Thus, research efforts have focused on improving the cultivation techniques, strain selection, and downstream processing methods to enhance the efficiency and cost-effectiveness of microalgal biotechnology for biofuel production. The development of genetically modified microalgae with enhanced lipid productivity and stress tolerance through targeted genetic modifications has already been demonstrated [16]. Genetic strain engineering of microalgae is a promising solution for the maximal lipid production. However, regulatory constraints and potential ecological impacts need to be addressed. Another novel strategy involves the development of photobioreactor systems designed to maximize the lipid production. Advanced photobioreactors offer precise control over environmental parameters such as the light intensity, temperature, and nutrient availability, optimizing microalgal growth and lipid accumulation [17]. Despite their potential, photobioreactor systems may pose challenges regarding the initial investment costs, the maintenance requirements, and their scalability for large-scale production. Recent efforts have focused on wastewater-based cultivation systems that utilize nutrient-rich wastewater streams as the growth media for the microalgae, simultaneously mitigating environmental pollution and reducing the cultivation costs [18]. While this approach offers ecological benefits and cost savings, the treatment efficacy, contaminant removal, and regulatory compliance must be addressed.

Co-cultivation to enhance the production of biofuels and lipids is gaining rapid interest in microalgal biotechnology [19]. Co-cultivation involves cultivating different microorganisms together in the same growth conditions, capitalizing on their synergistic interactions to improve the biomass and lipid contents [20,21]. Co-cultivation can potentially increase the biomass yield and total lipid yield compared to those in monoculture systems. Co-cultivation can enhance the overall productivity by harnessing the complementary metabolic capabilities and nutrient crosstalk of different species. Co-cultivation of two microalgal strains has resulted in a higher lipid productivity compared to that of monoculture, highlighting the potential of this approach for biofuel production [22]. Co-cultivation with specific bacterial strains of *Bacillus subtilis* significantly increased the lipid content in microalgae [23], and co-cultivation with the bacteria *Azospirillum brasilense* enhanced nutrient cycling and bioavailability. Bacteria can contribute to nutrient remineralization, nitrogen fixation for nitrogen-fixing cyanobacteria, and organic matter degradation, which can enhance the growth and productivity of microalgae [24]. The co-cultivation of microalgae with *Pseudomonas putida* facilitated the degradation of inhibitory compounds in microalgae cultures, leading to enhanced growth and lipid accumulation [25]. *Rhizobium* spp. enhanced the nutrient cycling and bioavailability in microalgae cultures, suggesting their role in optimizing the nutrient utilization efficiency and promoting lipid biosynthesis [26]. The co-cultivation of microalgae with *Chryseobacterium* spp. enhanced the lipid accumulation in microalgae through the secretion of growth-promoting metabolites. This highlights the potential of *Chryseobacterium* spp. to facilitate lipid synthesis pathways and improve lipid productivity [27]. However, maintaining stable co-culture ratios over long cultivation periods can be challenging, as competitive interactions or species dominance may occur. Finding compatible microalgal strains and optimizing the cultivation conditions for co-culture systems can achieve the maximal outcomes. The scalability, economic feasibility, and complexity of managing co-cultivation systems for large-scale biofuel production may affect the cost-effectiveness of this approach. Research efforts are thus needed to optimize the co-cultivation strategies, identify suitable microalgal combinations, and develop scalable cultivation technologies [19].

Hence, we offer immobilization, a widely applied technique in biotechnology, to develop a novel co-cultivation bioprocess for lipid and 2,3-butanediol production, with the co-cultivation of a plant-growth-promoting rhizobacterium and microalga. In our earlier publication, we already established the advantages of biopolymeric immobilization of the plant-growth-promoting rhizobacterium *Paenibacillus polymyxa* for biofuel production [28]. We developed a novel co-cultivation bioprocess with an immobilized plant-growth-promoting bacterium *Paenibacillus polymyxa* and the microalga *Scenedesmus obliquus* for lipid production, leveraging their synergistic interactions to optimize the biomass productivity and lipid content. Immobilization with biopolymers like chitosan and κ-carrageenan also has an added advantage in that carrageenan can interact with plasma membrane receptors using a co-receptor involved in signal transduction, leading to the simultaneous activation of plant growth [29]. Plant growth is regulated by various metabolic processes, such as photosynthesis, cell division, the purine and pyrimidine synthesis pathways, and the metabolic pathways involved in nitrogen and sulphur assimilation [30,31,32]. In addition, the production of secondary metabolites, signalling, and defence gene expression are induced in plants. For example, microalgae cell walls can be strengthened by forming structural proteins [33]. In addition, κ-carrageenan has been reported to increase the biomass concentration by 48%, and κ-carrageenan can be utilized for plant growth [31]. The effect of chitosan and κ-carrageenan has only been investigated in terms of plant growth [29,34,35]. There are no studies on the transferability of these effects to microalgae as a solution and an immobilization material. This research provides evidence of the effect of κ-carrageenan on microalgal growth. A fixed bed photoreactor was further constructed, and this immobilized co-cultivation bioprocess was implemented for improved and consistent bacterial 2,3-butanediol and microalgal lipid production.

This study aimed to develop and evaluate a novel co-fermentation process utilizing the chemical crosstalk between the freshwater microalga *Scenedesmus obliquus* and the biopolymer-immobilized plant-growth-promoting bacterium *Paenibacillus polymyxa* for the efficient and sustainable production of valuable metabolites in biotechnology applications.

## 2. Materials and Methods

### 2.1. The Preculture and Main Culture

The preculture and primary culture of *Paenibacillus polymyxa* ATCC 842 and *Scenedesmus obliquus* SAG 276-1 from DSMZ, the German Collection of Microorganisms and Cell Cultures GmbH, Braunschweig, Germany were aseptically cultivated, as described by Joshi et al. [28]. The pre-culture inoculated with 3 mL of the *S. obliquus* culture strain in 27 mL BG-11 medium [36] (Supplementary Materials) was cultivated on a self-constructed light shaker with an illuminance of 670 μmol/m^2^/s, using 75 W 24 VDC LED lights (Pur-Led Technik, Undenheim, Germany) and a shaking frequency of 120 rpm at 26 °C. The inoculation volume of the main cultures was calculated so that the microalgae had an initial cell concentration of 1 × 10^6^ cells per mL. For co-cultivation with the encapsulated bacteria, the initial cell concentration in the microcapsule was calculated so that it was 1 × 10^7^ cells per mL. For the cultivation of the microalgae with empty beads, 4 mL of 2.5% (weight/volume) carrageenan, which corresponded to 40 beads, was added to the medium. For the co-culture, 100 μL of 1 × 10^6^ cells per mL of *P. polymyxa* cells was immobilized with 4 mL of 2.5% (weight/volume) carrageenan solution. This were then added to 26.9 mL of PS medium with 3 mL of *S. obliquus*. The total volume of all of the cultures reached 30 mL with the *Paenibacillus–Scenedesmus* (PS) medium (Supplementary Materials) in 5 replicates.

#### 2.1.1. Chitosan-Coated Carrageenan Beads

A complex coacervation method was applied to forming the chitosan-coated carrageenan beads, as described by Joshi et al., 2025 [28].

#### 2.1.2. Chitosan-Coated Calcium Alginate Beads

Ionotropic gelation was applied to forming the chitosan-coated calcium alginate beads, as described by Joshi et al., 2025 [28].

### 2.2. The Cell Count

The cell count was determined with the help of a Bürker counting chamber, as described by Joshi et al., 2025 [28].

### 2.3. Substrate Analysis

For the 2,3 butanediol (2,3-BDL) and glucose concentrations, 1 mL samples were removed during each cultivation and frozen at −28 °C. Determination was carried out using an HPLC (high-performance liquid chromatography) system. The materials were separated using a reverse-phase column (column: Repromer H, 9 μm, 250 × 8 mm, Altmann Analytik, Munich, Germany). The analysis was performed as described by Joshi et al., 2025 [28].

### 2.4. Growth of the Microorganisms

The specific growth rate (μ) describes the increase in the cell concentration in the growth phases. Furthermore, the specific substrate uptake speed (qS) and product formation rate (qP) analogous to the growth rate were calculated (Supplementary Materials) as described by Joshi et al., 2025 [28].

### 2.5. Biomass Concentration

The dry biomass was determined gravimetrically in triplicate. At the end of the cultivation, 1 mL of the cell suspension was poured into a 1.5 mL pre-dried and balanced reaction vessel. After centrifugation of the sample at 14,000 rpm for 5 min, the supernatant was carefully decanted. The cell pellet was then dried in a 60 °C drying cabinet for 24 h. After drying, the reaction vessel was weighed. To determine the biomass concentration of the algal cells in co-cultivation with the immobilized bacteria, each 3 mL cell suspension was pre-weighed using a filtered cellulose nitrate membrane filter (Sartorius AG, Göttingen, Germany) with a pore size of 5 µm, sorting both microorganisms according to size. Algae cells remain on the membrane filter, and the bacteria remains in the filtrate. The membrane filter was dried in a 60 °C drying cabinet for 6 h and then weighed. The biomass concentration was calculated as follows:

(1) c_x=((m_full−m_empty)/V,

where m_full represents the mass of the dried reaction vessel or the membrane filter with the cell pellet [g], m_empty represents the mass of the dried empty reaction vessel or the membrane filter [g], and V represents the volume [L].

### 2.6. Lipid Content

Microalgae lipids were analyzed using a modified version of the method of Salama et al. [37]. Determination was carried out gravimetrically in triplicate. A 10 mL cell suspension was taken at the end of cultivation and centrifuged at 4500 rpm for 10 min. The supernatant was carefully decanted, and the cell pellet was then dried in a 50 °C cabinet for 6 h. A 0.1 g dried cell pellet was placed in a pre-dried, weighed 10 mL screw-threaded glass centrifuge tube. For extraction, 1.25 mL of chloroform and 2.5 mL of methanol were added. The sample was then sonicated at the maximum intensity for 30 min. This was followed by overnight incubation at 27 °C with shaking at 100 rpm. The next day, 1.25 mL of chloroform was added, and the extraction mixture was sonicated for another 30 min. After that, 1.25 mL of double-distilled water (ddH_2_O) was added, and the solution was centrifuged off for 10 min at 4500 rpm. A two-phase system was visible, with the upper phase containing the lipids. Through filtration of the lipid-rich phase using a membrane filter (Sartorius AG, Göttingen, Germany) made of PTFE with a pore size of 0.2 μm, the filtrate was able to be pre-weighed and was then transferred into another centrifuge tube. The chloroform was evaporated at room temperature under a vapor hood overnight. The next day, the sample was dried in a 50 °C drying cabinet for 24 h. Finally, the weight of the centrifuge tube was determined. The lipid content (LC) and the lipid yield (LY) were calculated using the following formulas:

LC (%) = (Wb − Wa)/Wo × 100%, (2)

LY (g/L) = (DWi − DWo) × LC, (3)

where Wo represents the empty weight of the centrifuge tube [g], Wb represents the weight of the centrifuge tube after extraction [g], Wa represents the weight of the weighed cell pellet [g], DWo represents the biomass concentration at time t0 [g/L], and DWi represents the biomass concentration at time ti [g/L].

### 2.7. Determination of the Pigment Composition

Determination of the pigment composition in the algae cells was carried out using solvent extraction. For this purpose, 200 μL of the sample to be determined was mixed with 800 μL of 100% methanol and incubated for 1 h at 65 °C in a water bath (Witeg Labortechnik, Wertheim am Main, Germany) with a closed lid to avoid any light reaction. Then, the sample was centrifuged off at 15,000 rpm for 5 min at 18 °C. The supernatant was poured into a precision cuvette made of quartz glass (Hellma Analytics, Müllheim, Germany) with a path length of 1 cm. For carotenoid and chlorophyll (Chl) determination, the absorbance was measured at 645 nm (A_645) and 663 nm (A_663) or at 470 nm (A_470) in a Genesys 10S UV-Vis Bio spectrophotometer, Thermo Scientific, Karlsruhe, Germany. The measurements of the samples were taken against 80% methanol as a reference. The chlorophyll a (Chla), chlorophyll b (Chlb), carotenoid, and total chlorophyll levels were calculated using the following formulas [38].

(4) Chl a(g/L)=[(A_663×0.0127)−(0.00269×A_645)]×Dilution factor,

(5) Chl b(g/L)=[(A_645×0.0229)−(0.00468×A_645)]×Dilution factor,

(6) Total of chlorophyll(g/L)=Chl a+Chl b,

where A represented the absorbance, and the Chl a and Chl b values calculated from the equations were applied in the following equation to determine the carotenoid content [34].

(7) Carotenoid(g/L)=(1×A_470−0.00227(Chl a)−0.0814(Chl b))/0.227

### 2.8. Photobioreactor Construction

**Figure 1:**
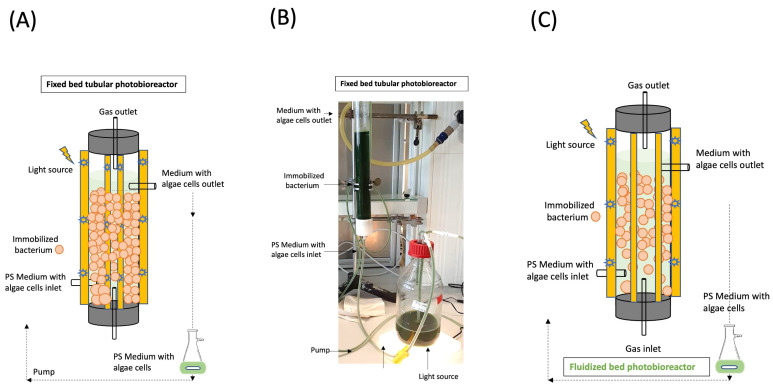
Setup of the utilized self-constructed fixed bed photobioreactor (**A**) and a photo of the utilized self-constructed tubular fixed bed photobioreactor with the main light source of the tubular photobioreactor removed to visualize the medium (**B**). Fluidized bed photobioreactor can improve the product yields with proper mixing due to gas sparging and improved light transmission (**C**).

Figure 1 presents the setup of the self-constructed fixed bed photobioreactor. It was a simplified setup with a transparent 50 × 4 cm length × diameter glass column (Duran Group, Mainz, Germany) filled with chitosan-coated carrageenan bacteria beads without mixing and no external gas supply, an illuminance of 670 μmol/m^2^/s, 75 W 24 VDC LED lights (Pur-Led Technik, Undenheim, Germany), silicon 100×0.5 cm length × diameter tubes, a mini pump (CBS Scientific, San Diego, CA, USA), stainless steel clamps, and a sterile 1 L borosilicate bottle (VWR International GmbH, Darmstadt, Germany) with PS medium and the axenic microalgae at room temperature, 27 °C, and the medium was fed at a flow rate of 0.2 mL/min. The calculated hydraulic retention time was 2.27 days, and the total period of cultivation was 336 h with 12 h sampling.

### 2.9. Experimental Designs and Statistical Analyses

Each experiment was carried out with 5 replicates unless specified. The statistical analyses were performed using IBM SPSS software, version 28. For data on the growth, the homoscedasticity of the data was tested and then analyzed first using a one-way ANOVA and then through a least significant difference (LSD) post hoc analysis. Significant differences in the figures are indicated by the letters a, b, and c. The values for p and n are denoted in the respective figure or table descriptions.

## 3. Results

### 3.1. Co-Cultivation with Immobilized Bacteria Showed Increased Growth and Total Chlorophyll Content in the Microalgal Cells

**Figure 2:**
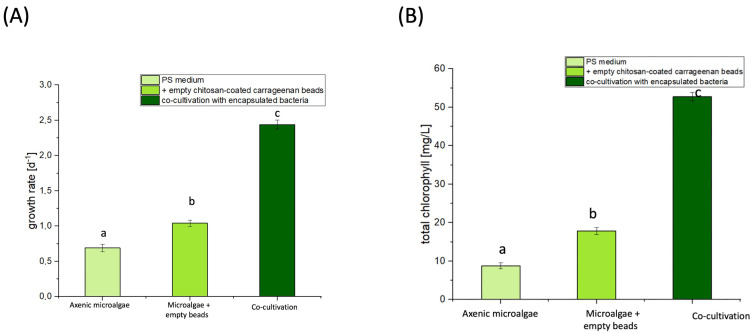
Maximum specific growth rates (μmax) determined in the cultivation of axenic microalgae in the PS medium with the addition of empty chitosan-coated carrageenan beads and the co-cultivation with immobilized bacteria. *n* = 5; mean ± SD. Different letters a, b, and c indicate a significant difference according to the one-way ANOVA F_2,14_ = 1501.51; *p* < 0.001 with Bonferroni’s post hoc test at *p* < 0.05. *n* = 5; mean ± SD; one-way ANOVA with Bonferroni’s post hoc test, *p* < 0.05 (**A**). Total chlorophyll contents determined in the cultivation of axenic microalgae in the PS medium with the addition of empty chitosan-coated carrageenan beads and the co-cultivation with immobilized bacteria. *n* = 5; mean ± SD. Different letters a, b, and c indicate a significant difference according to the one-way ANOVA F_2,14_ = 2894.11; *p* < 0.001 with Bonferroni’s post hoc test at *p* < 0.05 (**B**).

The results from Figure 2 and Table 1 demonstrate that both carrageenan and co-cultivation significantly enhanced the growth of *S. obliquus* with *P. polymyxa*, a plant-growth-promoting rhizobacterium (PGPR). The plant-growth-promoting properties of *P. polymyxa*, as well as the growth effect of carrageenan, were demonstrated.

Figure 2A illustrates the maximum specific growth rate (µmax) of the microalgae cultivated under various conditions, demonstrating the beneficial effects of the biopolymers and their application to the immobilized bacteria. Co-cultivation with the bacteria immobilized using the coated beads showed increased growth rates and total chlorophyll contents in the microalgae. The μ_max_ of the cultivation with the empty carrageenan beads was 0.94 ± 0.08/d with a maximal cell concentration of 13 × 10^7^ cells/mL. When cultivating using the empty chitosan-coated carrageenan beads, a doubling time of 0.69 ± 0.03 d was observed. During co-cultivation, a maximal cell concentration of 20.7 × 10^7^ cells/mL and a μ_max_ of 2.44 ± 0.06/d were determined, which was 3.5 times higher than that of the axenic microalgae. Comparing the μ_max_ in the cultivation of the axenic microalgae with the empty chitosan-coated carrageenan beads, a 2.4-fold increase was observed. Comparing the maximal cell concentration of 6.27 × 10^7^ cells/mL in the cultivation of the axenic microalgae, a 60.5% increase was observed with co-cultivation. The statistical analysis confirmed these differences as significant (one-way ANOVA F_2,14_ = 1501.51; *p* < 0.001 with Bonferroni’s post hoc test at *p* < 0.05). Figure 2B illustrates the total chlorophyll content in the axenic microalgae cultivation, cultivation with empty chitosan-coated carrageenan beads, and the co-cultivation with immobilized bacteria in the PS medium. The addition of the empty chitosan-coated carrageenan beads resulted in a total chlorophyll content of 17.9 ± 0.97 mg/L, representing a statistically significant 2-fold increase compared to that in the axenic cultures (one-way ANOVA F_2,14_ = 2894.11; *p* < 0.001 with Bonferroni’s post hoc test at *p* < 0.05). The co-cultivation with the immobilized bacteria yielded a total chlorophyll content of 52.7 ± 1.17 mg/L, which was 3-fold higher than that in the cultivation of axenic microalgae with the empty chitosan-coated beads. This increase was also statistically significant according to the one-way ANOVA (F_2,14_ = 2894.11; *p* < 0.001 with Bonferroni’s post hoc test at *p* < 0.05). Table 1 demonstrates that the chlorophyll content produced per cell was also found to be highest in the co-cultivation with the immobilized bacteria, suggesting that co-cultivation with the bacteria indeed aided the microalgae. These results suggest that both carrageenan and co-cultivation with *P. polymyxa* significantly increased the chlorophyll content of *S. obliquus*.

### 3.2. Co-Cultivation with the Immobilized Bacteria Improved the Biomass and Lipid Production by S. obliquus

The influence of carrageenan and co-cultivation on the biomass and lipid production by *S. obliquus* was investigated. The dry biomass concentrations (biomass concentrations), biomass-specific substrate uptake rates (qS), biomass yields (YX/S), lipid yields (LYs), and lipid contents (LCs) in the PS medium from the cultivation of the axenic microalgae, as well as these values with the addition of the empty chitosan-coated carrageenan beads and co-cultivation with the immobilized bacteria in the PS medium, are presented below.

Comparing co-cultivation with axenic microalgae cultivation using the empty chitosan-coated carrageenan beads revealed a 3.3-fold increase in the biomass concentration, which was statistically significant (one-way ANOVA F_2,14_ = 4438.43; *p* < 0.001 with Bonferroni’s post hoc test at *p* < 0.05). Table 2 demonstrates that carrageenan did not significantly affect the substrate uptake rate in *S. obliquus* cultivation (one-way ANOVA F_2,14_ = 14.91; *p* = 1.000 with Bonferroni’s post hoc test at *p* < 0.05). However, it increased the biomass yield by 31%, which was statistically significant (one-way ANOVA with Bonferroni’s post hoc test at *p* < 0.05 (F_2,14_ = 179.79, *p* < 0.001 (YX/S)). Co-cultivation with the immobilized bacteria resulted in a 34.8% higher substrate uptake rate and a 77.6% higher biomass yield compared to these values in the *S. obliquus* axenic culture. These differences were statistically significant (one-way ANOVA with Bonferroni’s post hoc test at *p* < 0.05; F_2,14_ = 14.91, *p* < 0.001 (qS); F_2,14_ = 179.79, *p* < 0.001 (YX/S)).

Table 3 demonstrates that co-cultivation achieved the highest lipid concentration at 3.46 ± 0.11 g/L. Comparing co-cultivation with axenic microalgae cultivation using the empty chitosan-coated carrageenan beads revealed a 2.3-fold increase in the lipid concentration, which was statistically significant (one-way ANOVA with Bonferroni’s post hoc test, F_2,14_ = 4438.43; *p* < 0.001). Among all the cultivations, co-cultivation yielded the highest lipid content at 32%. Carrageenan alone significantly increased the lipid content by 2.3 times, while co-cultivation with the immobilized bacteria enhanced it by 54.9%. The statistical analysis confirmed significant differences in the lipid contents (one-way ANOVA F_2,14_ = 782.71; *p* < 0.001 with Bonferroni’s post hoc test at *p* < 0.05). Both carrageenan and co-cultivation with *P. polymyxa* substantially increased the lipid content compared to that in axenic microalgae cultivation, with 2.3-fold and 3.6-fold increases, respectively (one-way ANOVA F_2,14_ = 782.71; *p* < 0.001 with Bonferroni’s post hoc test at *p* < 0.05). These results further demonstrate the growth effect of carrageenan.

### 3.3. Co-Cultivation with the Microalgae Improved the 2,3-Butanediol Production by Immobilized P. polymyxa

The influence of immobilized co-cultivation on the 2,3-butanediol (2,3-BDL) production by the immobilized *P. polymyxa* was investigated. The co-cultivation was compared with that for *P. polymyxa* immobilized in two different biopolymeric formulations.

**Figure 3:**
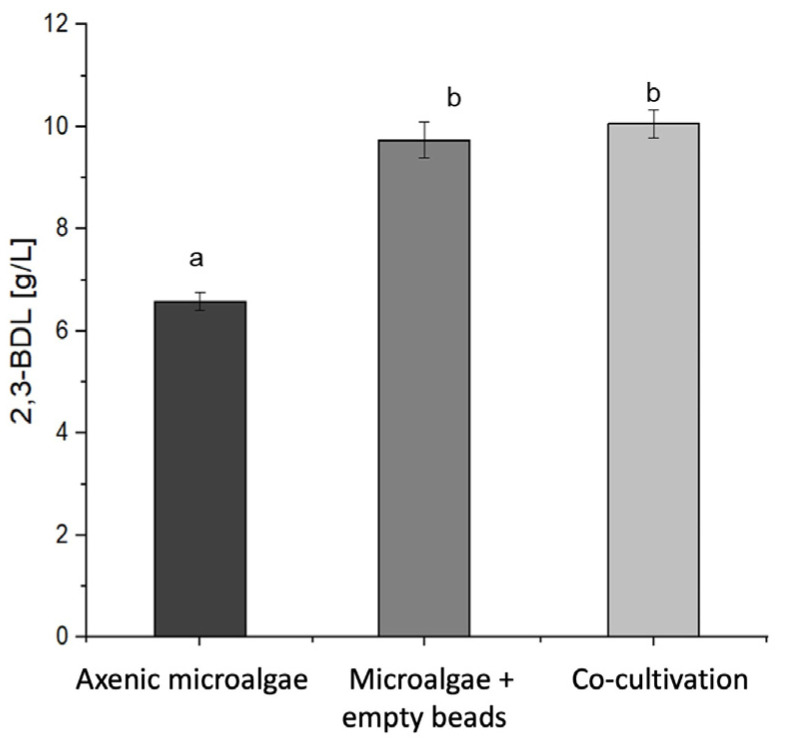
Determined 2,3-BDL concentrations of co-cultivation of free cells and chitosan-coated calcium alginate bead- and chitosan-coated carrageenan bead-immobilized bacteria. *n* = 5; mean ± SD. Different letters a and b indicate a significant difference according to one-way ANOVA F_2,24_ = 166.58; *p* < 0.001 with Bonferroni’s post hoc test at *p* < 0.05.

Figure 3 illustrates that the co-cultivation with the chitosan-coated carrageenan bead-immobilized bacteria achieved a product formation of 10.06 ± 0.23 g/L of 2,3-BDL, while co-cultivation with the chitosan-coated calcium alginate-immobilized bacteria produced 9.55 ± 0.28 g/L of 2,3-BDL. The carrageenan beads enabled a 5.3% higher 2,3-BDL production compared to that with the alginate beads, though this increase was not statistically significant (one-way ANOVA F_2,24_ = 166.58; *p* = 1.000 with Bonferroni’s post hoc test at *p* < 0.05). The immobilized bacteria significantly outperformed the free cells in terms of 2,3-BDL production. The calcium alginate and carrageenan beads achieved 45.1% and 52.9% higher 2,3-BDL concentrations compared to those achieved using the free cells. These differences were statistically significant at *p* < 0.05 according to the one-way ANOVA with Bonferroni’s post hoc test (F_2,24_ = 166.58; *p* < 0.001). Table 4 summarizes the product formation rates (qP) and product yields (YP/S) for each cultivation. Overall, co-cultivation of the microalgae with the immobilized bacteria increased the 2,3-butanediol production as compared to that with the axenic bacteria.

Table 4 demonstrates no significant difference in the product formation rate or yield between the two bead systems (one-way ANOVA F_2,24_ = 0.62, *p* = 1.000 (qP); F_2,24_ = 0.62, *p* = 1.000 (YP/S) with Bonferroni’s post hoc test at *p* < 0.05). The product formation rates of co-cultivation with the immobilized bacteria showed no significant difference compared to those for the free cells (one-way ANOVA F_2,24_ = 0.62; *p* = 0.547 with Bonferroni’s post hoc test at *p* < 0.05). However, co-cultivation with the immobilized bacteria yielded a higher 2,3-BDL production than that with the free cells. Specifically, co-cultivation with the calcium alginate beads resulted in a 36.2% higher 2,3-BDL yield while the carrageenan beads achieved a 42.6% increase. A statistical analysis using a one-way ANOVA showed a significant difference in the product yield (F_2,24_ = 39.94, *p* < 0.001 with Bonferroni’s post hoc test at *p* < 0.05). The enhanced 2,3-BDL yields observed in co-cultivation with the immobilized bacteria can be attributed to the physical separation of the bacteria from microalgae, which likely stimulated growth and secondary metabolite production. This immobilization strategy prevented direct contact between the two organisms while allowing for beneficial interactions, potentially through the exchange of metabolites or signaling molecules. These findings highlight the effectiveness of bacterial immobilization in co-cultivation systems in improving 2,3-BDL production, with both beads showing promise as immobilization matrices.

### 3.4. The Implementation of a Novel Co-Cultivation Bioprocess for Lipid and 2,3-Butanediol Production Through Co-Cultivation of an Immobilized PGPR and Microalga for Combined Beneficial Effects

The co-cultivation of axenic *S. obliquus* with *P. polymyxa* immobilized in chitosan-coated carrageenan beads in PS medium was implemented in a self-constructed fixed bed photobioreactor. The cultivation medium and conditions were similar to those in the shake flask experiments. Comparison with shake flask cultivation revealed that the photobioreactor enabled higher cell concentrations, possibly due to the reduced stress due to contamination and shear forces, resulting in a more successful cultivation process.

**Figure 4:**
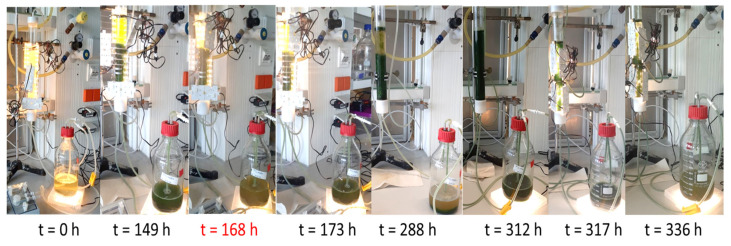
Co-cultivation of axenic *S. obliquus* with *P. polymyxa* immobilized in chitosan-coated carrageenan beads in PS medium using a self-constructed tubular fixed bed photobioreactor over 366 h; photos with the main light source of the tubular photobioreactor removed to visualize the medium.

**Figure 5:**
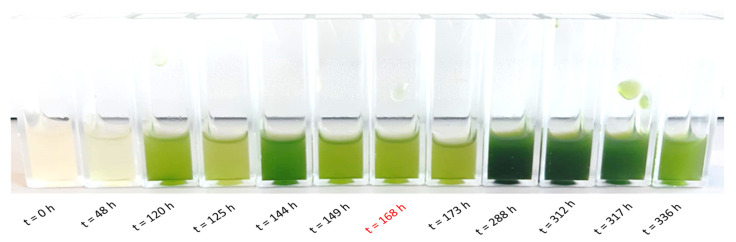
Samples of *S. obliquus* in co-culture with chitosan-coated carrageenan *P. polymyxa* beads in PS medium in a photobioreactor for 0 h to 336 h.

Table 5 demonstrates a comparative analysis of both cultivation systems focusing on the specific growth rate and dry biomass. The photobioreactor resulted in a 1.68 h faster maximum specific growth rate with a doubling time (t_d_) of 0.84 d as compared to 0.91 d with shake flask cultivation. The dry biomass and the substrate uptake rate were also slightly higher for the photobioreactor cultivation at 12.50 ± 0.26 g/L and 24.99 gSubstrate/gBiomass.time (gS/gX·d) compared to 11.79 ± 0.16 g/L and 23.59 gS/gX·d in the flask. The biomass yield remained constant for both cultivations at 0.03 gX/gS. Figure 4 and Figure 5 depict the co-cultivation of axenic *S. obliquus* with *P. polymyxa* immobilized in chitosan-coated carrageenan beads in PS medium using a self-constructed tubular fixed bed photobioreactor over 366 h.

These results highlight the potential advantages of using a fixed bed photobioreactor for the co-cultivation of microalgae and immobilized bacteria, particularly regarding the growth rate and biomass production. The consistency of the biomass yield suggests that the improved growth conditions in the photobioreactor did not compromise efficiency of the conversion of biomass from the substrate. Further research into photobioreactor and bioprocess optimization will establish a robust large-scale microalgal production process.

## 4. Discussion

Microalgae biotechnology has become increasingly important due to its potential for high biomass production and the accumulation of lipids and carbohydrates [6,7,8]. However, low growth rates, expensive media, and contamination risks have hindered its economic viability. Recent studies have shown that co-cultivation with bacteria can improve microalgae processes through symbiotic effects due to the exchange of carbon dioxide and other nutrients [39,40,41,42] potentially enhancing the photosynthetic efficiency [43] and lipid content [44,45]. This co-culture approach offers increased biomass and lipid yields and enhanced chlorophyll content and secondary metabolites [46,47]. Immobilization techniques have been developed to avoid mutual inhibition through direct contact [48]. Various polymers are utilized, with κ-carrageenan being particularly successful due to its simple formulation, mild gelling conditions, low toxicity, and ability to act as a bioactive compound, stimulating plant growth. Co-culture systems present a promising strategy for addressing green energy contamination problems in microalgae cultivation, which has traditionally focused on monoculture systems [49]. Microalgae–bacteria exchange various nutrients and growth-triggering molecules in co-cultivation via commensalism and mutualism [50,51,52]. This profitable symbiosis involves intricate nutrient recycling, nitrogen fixation, carbon assimilation, oxygen production, and the exchange of dissolved organic matter [53,54,55,56]. Additionally, the exchange of siderophores and vitamins further enhances this mutually beneficial interaction [57,58]. Selecting strains with a high lipid productivity and compatible nutrient profiles to minimize the competition in co-cultivation systems and overcoming challenges in scaling up these processes will further this research [53,59].

This study aimed to examine the impact of immobilization and co-cultivation on the lipid and pigment production from *S. obliquus* and the 2,3-BDL production by *P. polymyxa*. For this purpose, both free and immobilized bacteria were cultivated in mono- and co-cultures. The influence of immobilization on the growth of *P. polymyxa* in co-cultivation with *S. obliquus* was then investigated using two types of bead systems [28]. It was found that the maximum cell concentrations were reached earlier during co-cultivation and the lag phase was thus shortened. The positive influence of the microalgae on the bacterial growth was demonstrated, and the co-cultivation of the immobilized bacteria with the microalgae increased the cell concentration of the bacteria. The co-cultivation of immobilized bacteria with microalgae promoted lipids and 2,3-BDL production.

Challenges with microalgal processes can be overcome through their co-cultivation with plant-growth-promoting bacteria [45,59]. In addition to co-cultivation, this study examined the effect of chitosan and κ-carrageenan on the microalgae, which have proven to be effective growth inducers in plants [29,30,34,35]. For this purpose, cultivation of *S. obliquus* with the addition of carrageenan in bead form was carried out in mono- and co-cultures, as shown in [28]. The effect of carrageenan, which is an effective growth inducer in plants, was thus demonstrated in *S. obliquus*. In addition, the effect of chitosan, which has also been established as a growth inducer in plants [60], on the microalgae was tested. Previous reports have shown that chitosan can improve plant photosynthesis [61], and hence chitosan was also employed in the coating of the beads. Consequently, it could be shown that the microalgae in the co-culture benefited from the bacteria due to carbon dioxide and other nutrients. In addition, the influence of carrageenan and co-cultivation with the immobilized bacteria on the biomass and lipid production by *S. obliquus* was investigated. When cultivating with empty chitosan-coated carrageenan beads, a 2.1 times higher biomass concentration was achieved which was significant, than the axenic cultivation. The lipid yield was also increased 4.9-fold by using carrageenan. Thus, the growth effects of carrageenan on *S. obliquus* could be demonstrated. Co-cultivation with the immobilized bacteria yielded a biomass concentration of 11.8 ± 0.51 g/L. This was 61.6% higher than that of the axenic microalgae with the empty chitosan-coated carrageenan beads. In addition, the lipid yield was increased by 2.3 times to 3.46 ± 0.11 g/L in co-cultivation. Therefore, it was confirmed that co-cultivation with the immobilized bacteria increased the biomass concentration and lipid production of the microalgae. Finally, the influence of carrageenan on the chlorophyll content of *S. obliquus* was investigated. With the addition of carrageenan, an 80.9% increase in the total chlorophyll content was recorded. Thus, the property of carrageenan being able to increase the chlorophyll content was also demonstrated for the microalgae. These results concurred that κ-carrageenan increased the chlorophyll content in the microalgae. When examining the influence of co-cultivation on the pigment content, a total chlorophyll content of 52.7 ± 1.17 mg/L was detected. Thus, the total chlorophyll content was increased by 20.4% and 3-fold, respectively, through co-cultivation. Overall, the growth of *S. obliquus* was greatly improved by both carrageenan and co-cultivation with *P. polymyxa*. Furthermore, the biomass and lipid yields, as well as the pigment contents, were increased. The plant-growth-promoting properties of *P. polymyxa* and the growth effect of carrageenan were thus demonstrated. Previous reports have shown that co-culturing *Synechococcus* sp. (cyanobacterium) beads with *Chlamydomonas reinhardtii* (microalgae) improved the growth and lipid production of the microalgae [45] and increased the possibility of recycling the beads [45,62]. Co-culturing *S. obliquus* and *Rhodotorula glutinis* in a 5 L photobioreactor reportedly generated a 40–50% increase and a 60–70% increase in the biomass and total lipid yields, respectively [63,64]. Co-culturing *Chlorella vulgaris* and *S. obliquus* and a *Chlorococcum* sp. resulted in an increase in the ethanol production in a 3 L photobioreactor at a light intensity of 4000 lux for 24 h of 58% for Juliarnita et al. [65]. Co-culture of yeast with immobilized *Chlorella vulgaris* microalgae produced the highest biomass of 12.2 g/L, with a high lipid content of 47% [48]. However, the co-cultivation of *P. polymyxa* and *S. obliquus* in our study is the first report wherein both of these microorganisms benefited each other in producing value-added products.

Scaling up microalgal–bacterial co-cultures from the laboratory to industrial levels often leads to significant bioproduct yield losses due to the unfavorable culture conditions or dead zones in photobioreactors [66,67]. Long-term operations are particularly prone to bacterial contamination, as bacteria with shorter doubling times can secrete algicidal toxins and cell-wall-degrading enzymes when their growth exceeds the optimal ratios. Overgrown bacterial communities obstruct light penetration, reducing microalgal biomass yields and lowering the production of light-dependent algal products like carotenoids and fatty acids due to the decreased photosynthetic efficiency [24]. This light limitation, compounded by high biomass concentrations, forces microalgae to consume stored lipids and carbohydrates for maintenance, further reducing the growth rates [68]. Immobilization of the cells on solid supports can mitigate these risks and contamination [69]. In this study, co-cultivation of *Paenibacillus polymyxa* with *Scenedesmus obliquus* was explored, with the bacteria encapsulated in chitosan-coated calcium alginate and carrageenan beads to prevent mutual inhibition. Using a fixed bed photobioreactor system, the process demonstrated advantages over traditional shake flask cultivation, including faster growth rates, higher dry biomass yields, and more efficient nutrient uptake. These improvements stemmed from the optimized light distribution, enhanced photosynthetic efficiency, and better gas and nutrient transfer in the photobioreactor. Despite these benefits, scaling up photobioreactors poses challenges such as higher construction and operational costs compared to those of open pond systems, the need for skilled operators, and potential difficulties in maintaining light penetration as the biomass increases [62,70]. Balancing bacterial and microalgal growth will hence require careful optimization. Immobilization technology enables higher biomass concentrations, which can lead to increased productivity and reduced production costs. Robust immobilization can lead to more stable production and potentially lower operational costs, as immobilization offers protection against shear forces, the prevention of overgrowth, and continuous product secretion, leading to low downstream processing costs for filtration and purification. The beads can also be recycled to optimize the immobilization material costs. The economic viability can be improved by integrating co-cultivation systems with other industrial processes, such as using flue gas for CO_2_ supply or combining them with wastewater treatment [70].

This novel co-cultivation bioprocess shows promise for enhancing lipid and 2,3-butanediol production through the synergistic interactions between immobilized plant-growth-promoting bacteria and microalgae. However, future research must address its scalability, long-term maintenance, and process optimization. Approaches should include strain screening, molecular-level studies of the co-culture interactions, metabolite exchange profiling, and developing advanced systems like fluidized bed photobioreactors to overcome the current limitations.

## 5. Conclusions

Co-cultivation is a promising solution for the biotechnological production of metabolites, wherein both organisms gain benefits from their interaction and chemical crosstalk. The synergistic effects observed between *P. polymyxa* and *S. obliquus*, coupled with the growth-promoting properties of immobilization biopolymer materials, offer sustainable bioproduction. This work contributes significantly to the growing knowledge on microbial co-cultivation strategies and their potential in biotechnological applications.

## Figures and Tables

**Table 1:** Co-cultivation with immobilized bacteria enhances chlorophyll production in microalgal cells.

Cultivation	Chlorophyll Content (mg/Cells)
a. axenic algae in PS medium	15 × 10^−6^
b. axenic algae with empty chitosan-coated carrageenan beads	18 × 10^−6^
c. co-cultivation with immobilized bacteria	26 × 10^−6^

**Table 2:** Determined biomass concentrations (g/L), biomass yields (YX/S), and biomass-specific substrate uptake rates (qS) in the different cultivations.

**Cultivation**	Biomass [g/L]	Biomass Yield [g_X_/g_S_]	Biomass-Specific Substrate Uptake Rates qS [gS/gXd]
a. axenic algae in PS medium	3.5 ± 0.31	0.58 ± 0.04	0.46 ± 0.06
b. axenic algae with empty chitosan-coated carrageenan beads	7.3 ± 0.54	0.76 ± 0.03	0.43 ± 0.04
c. co-cultivation with immobilized bacteria	10.8 ± 0.51	1.03 ± 0.02	0.62 ± 0.06

*n* = 5; mean ± SD. Significant differences according to one-way ANOVA for biomass concentration F_2,14_ = 309.43; *p* < 0.001, qS F_2,14_ = 14.91; *p* < 0.001 and YX/S F_2,14_ = 179.79; *p* < 0.001.

**Table 3:** Lipid yields (g/L) and lipid concentration of the various cultivations determined from the biomass concentrations.

Cultivation	Total Lipids [g/L]	Lipid Concentration [%]
a. axenic algae in PS medium	0.31 ± 0.09	8.86 ± 0.67
b. axenic algae empty chitosan-coated carrageenan beads	1.51 ± 0.04	20.68 ± 1.03
c. co-cultivation with immobilized bacteria	3.46 ± 0.11	32.04 ± 1.05

*n* = 5; mean ± SD. Significant differences according to one-way ANOVA with Bonferroni’s post hoc test at *p* < 0.05 for the biomass concentration F_2,14_ = 309.43; *p* < 0.001, LY F_2,14_ = 4438.43; *p* < 0.001 and LC F_2,14_ = 782.71; *p* < 0.001.

**Table 4:** Determined product formation rates (qP) and product yields (YP/S) of 2,3-BDL from co-cultivation of free cells and with immobilized bacteria.

Cultivation	Product Formation Rates qP [gP/gxd]	Product Yields YP/S [gP/gS]
a. Co-cultivation with free cells	0.15 ± 0.05	0.37 ± 0.02
b. Co-cultivation with chitosan–calcium alginate-immobilized bacteria	0.55 ± 0.07	0.54 ± 0.04
c. Co-cultivation with chitosan–carrageenan-immobilized bacteria	0.61 ± 0.55	0.57 ± 0.06

*n* = 5; mean ± SD. There were no significant differences according to one-way ANOVA with Bonferroni’s post hoc test at *p* < 0.05 for qP F_2,24_ = 0.62; *p* = 0.547. Significant differences for YP/S F_2,24_ = 39.94; *p* < 0.001.

**Table 5:** Maximum specific growth rate (μmax) and dry biomass concentration (g/L) of *S. obliquus* determined in co-culture with chitosan-coated carrageenan *P. polymyxa* beads in PS medium in a flask and in a photobioreactor.

Cultivation	Growth Rate µmax [d]	Dry Biomass Concentration [g/L]
Co-cultivation with BP-2 beads in PS medium (flask)	0.76 ± 0.02	11.79 ±0.16
Co-cultivation with BP-2 beads in PS medium (PBR)	0.83 ± 0.01	12.50 ± 0.26

## Data Availability

The data presented in this study are available upon request from the corresponding author. However, due to a cooperation agreement with the project partners, they are not publicly available.

## Supplementary Materials

The following supporting information can be downloaded at https://www.mdpi.com/article/10.3390/microorganisms13030606/s1. Figure S1: Self constructed light incubation shaker; Figure S2: Microscopic view of co-cultivation of *P. polymyxa* and *S. obliquus*; Figure S3: Cell concentrations of microalgae cultured in PS medium with the addition of empty carrageenan beads and chitosan-coated carrageenan beads against cultivation time; Figure S4: Cell concentrations of the cultivation of axenic microalgae in PS medium, with the addition of empty chitosan-coated carrageenan beads and the co-cultivation with encapsulated bacteria against cultivation time; Figure S5: Determined carotenoid contents of the cultivation of axenic microalgae in PS medium, with the addition of empty chitosan-coated carrageenan beads and the co-cultivation with encapsulated bacteria; Figure S6: Biomass concentrations of the cultivation of axenic microalgae in PS medium (A), with the addition of empty chitosan-coated carrageenan beads (B) and the co-cultivation with immobilized bacteria (C); Table S1: Paenibacillus Scenedesmus (PS) medium; Table S2: Trace elements; Table S3: BG-11 medium; Table S4: Metal Salt solution.
