# Supplementary material for: Novel Co-Cultivation Bioprocess with Immobilized Paenibacillus polymyxa and Scenedesmus obliquus for Lipid and Butanediol Production
Source: Microorganisms. 2025 Mar 5;13(3):606. doi: 10.3390/microorganisms13030606 (PMC11945626; doi:10.3390/microorganisms13030606)
Supplement: Supplementary file 1 [file microorganisms-13-00606-s001.zip › microorganisms-3479204-supplementary.pdf]

Supplementary Materials

Novel co-cultivation bioprocess of immobilized *Paenibacillus polymyxa* and *Scenedesmus obliquus* for lipid and butanediol production

Jnanada Joshi[1,2], Laura Fladung[1,2], Olaf Kruse[2] and Anant Patel[1*]

[1] Hochschule Bielefeld – University of Applied Sciences and Arts (HSBI), Germany; jnanada_shrikant.joshi@hsbi.de
[2] Bielefeld University, Germany; olaf.kruse@uni-bielefeld.de
* Correspondence: anant.patel@hsbi.de

2. Materials and Methods

2.1. Preculture and Main culture

Table S1: PaenibacillusScenedesmus (PS) medium

Component	Concentration [L^{-1}] (weight/volume)	Source of supply
Glucose	20g	Carl Roth GmbH & Co. KG, Karlsruhe, Germany
Yeast extract	5g	
Tryptone	5g	
$MgSO_4 \times H_2O$	0,2g	
$⟦(NH⟧_4)_2SO_4$	3g	
$KH_{\cdot 2}\,PO_{\cdot 4}$	3,5g	AppliChem GmbH, Darmstadt, Germany
$K_2HPO_{\cdot 4}$	2,75g	
Trace elements	90µL	see below

Table S2: Trace elements

Component	Concentration [L^{-1}] (weight/volume)	Source of supply
$FeSO_4$	40mg	Carl Roth GmbH & Co. KG, Karlsruhe, Germany
$H_3BO_{\cdot 3}$	80mg	AppliChem GmbH, Darmstadt, Germany
$CuSO_4 \times 5H_2O$	4mg	Carl Roth GmbH & Co. KG, Karlsruhe, Germany
$Na_2MoO_4 \times 2\,H_2O$	4mg	Merck KgaA, Darmstadt, Germany
$MnCl_2 \times 4\,H_2O$	500mg	
$ZnSO_4 \times 7\,H_2O$	10mg	Carl Roth GmbH & Co. KG, Karlsruhe, Germany
$⟦Co(NO⟧_3)_2 \times 6\,H_2O$	8mg	Merck KgaA, Darmstadt, Germany
$CaCl_2 \times 2\,H_2O$	100mg	

Biotin	1mg	Carl Roth GmbH & Co. KG, Karlsruhe, Germany

Table S3: BG-11 medium

Component	Concentration [L^{-1}] (weight/volume)	Source of supply
$NaNO_4$	1,5g	Carl Roth GmbH & Co. KG, Karlsruhe, Germany
K_2HPO_4	0,04g	AppliChem GmbH, Darmstadt, Germany
$MgSO_4 \times 7\ H_2O$	0,075g	Carl Roth GmbH & Co. KG, Karlsruhe, Germany
$CaCl_2 \times 2\ H_2O$	0,036g	
$Citric\ acid \times H_2O$	0,006g	VWR International GmbH, Darmstadt, Germany
Ammonium-|||-citrate	0,006g	Carl Roth GmbH & Co. KG, Karlsruhe, Germany
Na_2EDTA	0,001g	AppliChem GmbH, Darmstadt, Germany
Na_2CO_3	0,02g	Carl Roth GmbH & Co. KG, Karlsruhe, Germany
Metal salt solution	1mL	see below

Table S4: Metal salt solution

Component	Concentration [L^{-1}] (mL, weight/volume)	Source of supply
$H_3BO._3$	2,86	AppliChem GmbH, Darmstadt, Germany
$MnCl_2 \times 4\ H_2O$	1,81	Merck KgaA, Darmstadt, Germany
$ZnSO_4 \times 7\ H_2O$	0,22	Carl Roth GmbH & Co. KG, Karlsruhe, Germany
$Na_2MoO_4 \times 2\ H_2O$	0,39	Merck KgaA, Darmstadt, Germany
$CuSO_4 \times 5H_2O$	0,08	Carl Roth GmbH & Co. KG, Karlsruhe, Germany
$\llbracket Co(NO\rrbracket_3)_2 \times 6\ H_2O$	0,05	Merck KgaA, Darmstadt, Germany

(Stanier RY, Kunisawa R, Mandel M, Cohen-Bazire G. Purification and properties of unicellular blue-green algae (order Chroococcales). Bacteriol Rev. 1971 Jun;35(2):171-205. doi: 10.1128/br.35.2.171-205.1971. PMID: 4998365; PMCID: PMC378380.)

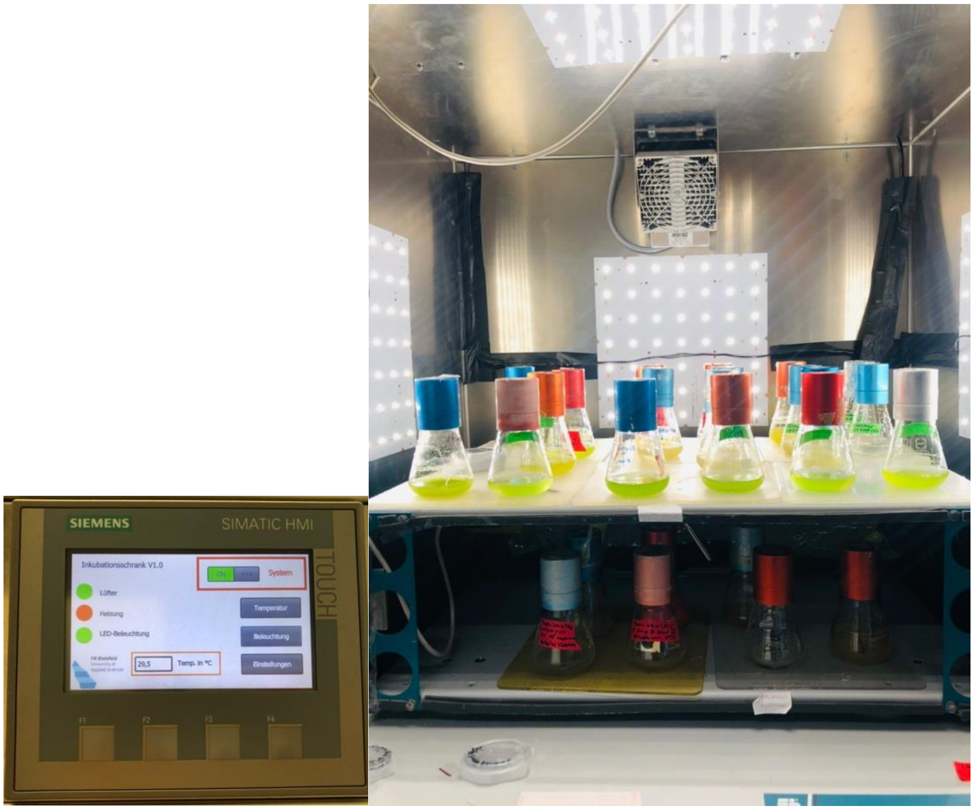

Figure S1. Self constructed light incubation shaker.

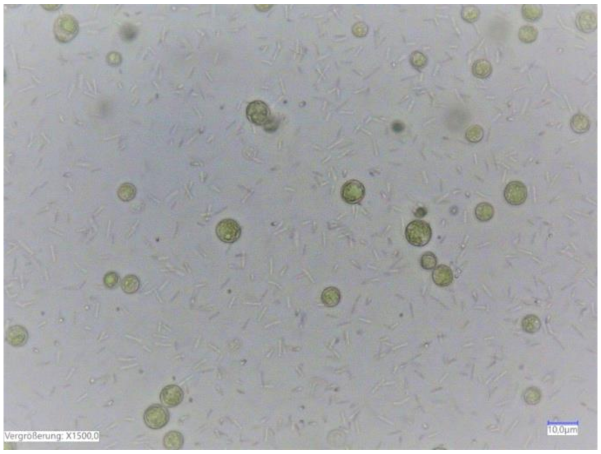

Figure S2. Microscopic view of co-cultivation of *P. polymyxa* and *S. obliquus*

3. Results

3.1 Co-cultivation with immobilized bacteria showed increased growth and total chlorophyll content in microalgal cells

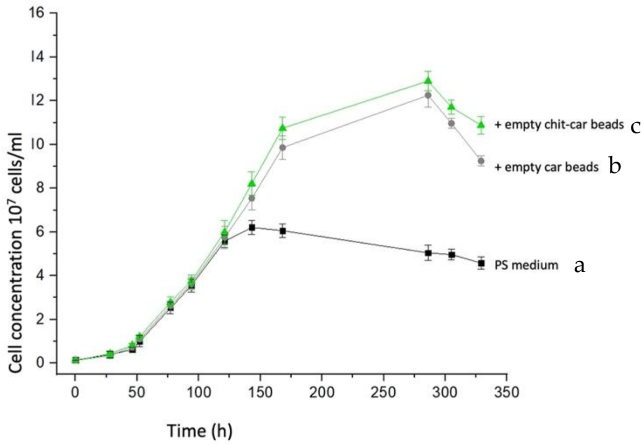

Figure S3. Cell concentrations of microalgae cultured in PS medium with the addition of empty carrageenan beads and chitosan-coated carrageenan beads against cultivation time. $n = 5$; mean ± SD. Different letters a,b,c indicate a significant difference according to RM-ANOVA $F_{2,12} = 202.09$; $p < 0.001$ with Bonferroni´s post-hoc test at $p < 0.05$.

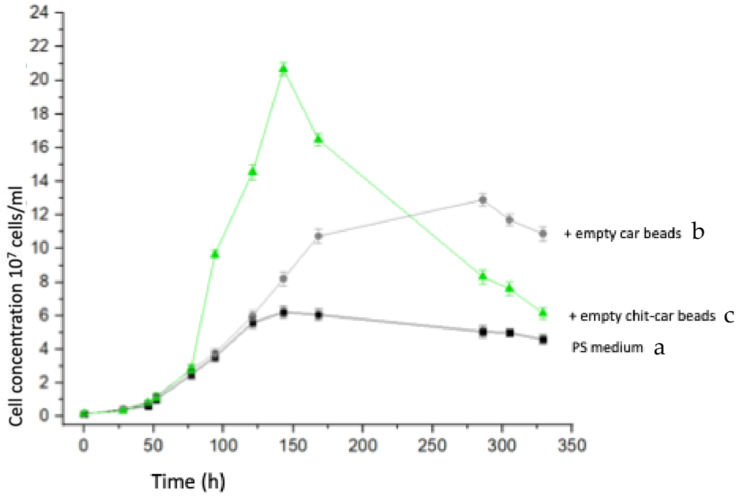

Figure S4. Cell concentrations of the cultivation of axenic microalgae in PS medium, with the addition of empty chitosan-coated carrageenan beads and the co-cultivation with encapsulated bacteria against cultivation time. $n = 5$; mean ± SD. Different letters a,b,c indicate a significant difference according to RM-ANOVA $F_{2,12} = 474.76$; $p < 0.001$ with Bonferroni´s post-hoc test at $p < 0.05$.

2.8. Determination of pigment composition

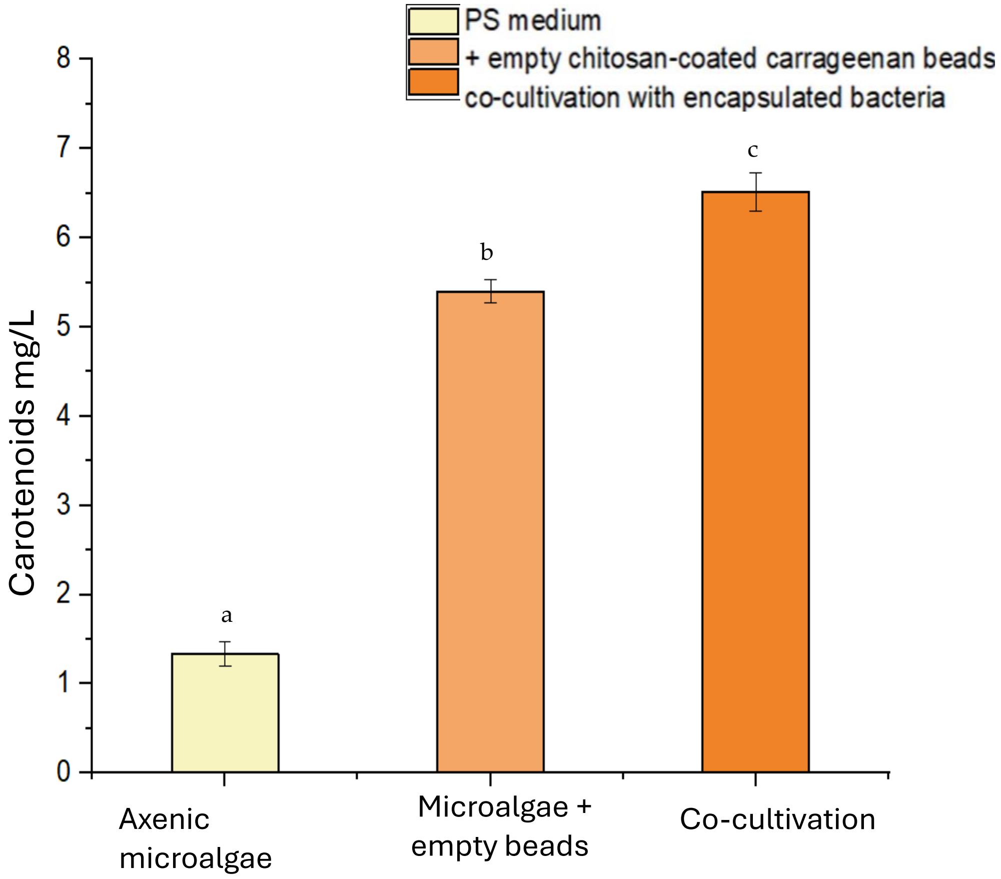

Figure S5. Determined carotenoid contents of the cultivation of axenic microalgae in PS medium, with the addition of empty chitosan-coated carrageenan beads and the co-cultivation with encapsulated bacteria. $n = 5$; mean ± SD. Different letters a,b,c indicate a significant difference according to one-way ANOVA $F_{2,14} = 1453.85$; $p < 0.001$ with Bonferroni´s post-hoc test at $p < 0.05$.

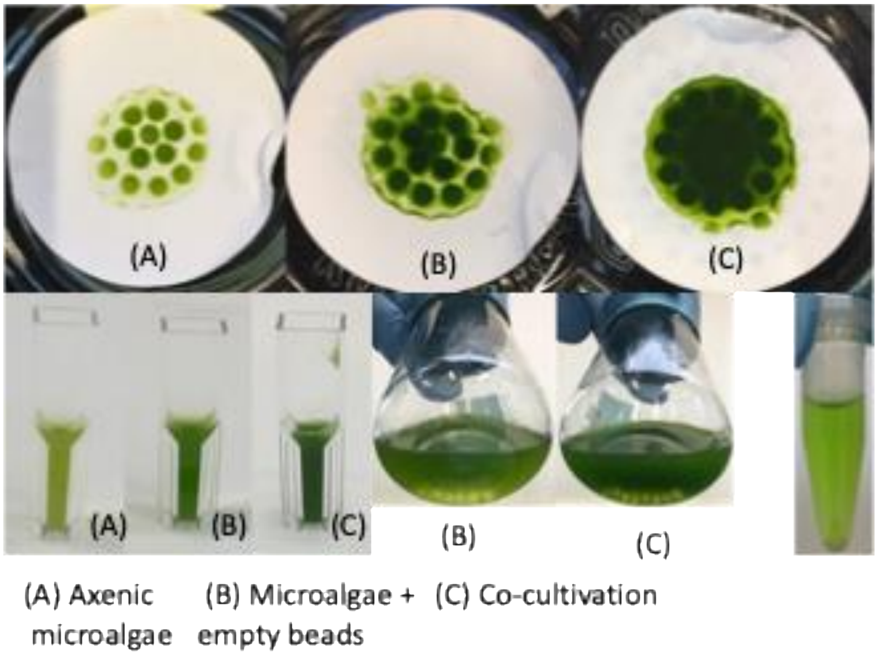

Figure S6. Biomass concentrations of the cultivation of axenic microalgae in PS medium (A), with the addition of empty chitosan-coated carrageenan beads (B) and the co-cultivation with immobilized bacteria (C)

- Growth of microorganisms

The specific growth rate μ describes the increase in cell concentration in the growth phases.
Furthermore, the specific substrate uptake speed q_S and product formation rate q_P analogous to the growth rate was described. The reaction rates for substrates were negative and positively defined for products. The following applies:

$$q_S = 1/x \; d_S/d_t \,, \qquad \text{(S1)}$$

$$q_P = 1/x \; d_P/d_t \,, \qquad \text{(S2)}$$

q_S represents the specific substrate uptake rate [g g-1 d-1], q_P represents the specific product formation rate [g g-1 d-1], S represents the substrate concentration [g L-1], P represents the product concentration [g L-1], X represents the biomass concentration [g L-1] and t represents the time [d]
For the mathematical representation of the product yield, $Y_{\frac{P}{S}}$ in relation to the supplied substrate concentration can be related to the corresponding set speeds.

$$Y_{(P/S)} = d_P/d_S = q_P/q_S \,, \qquad \text{(S3)}$$

$Y_{(P/S)}$ represents the product yield [g g^{-1}]

By applying the following equation, we can calculate the biomass yield per substrate consumed

$$Y_{(X/S)} = d_X/d_S = \mu/q_S \,, \qquad \text{(S4)}$$

$Y_{(X/S)}$ represents the biomass yield [g g^{-1}]
